# Reversal of Glenn-associated diffuse pulmonary arteriovenous malformations after total cavopulmonary connection: a case report

**DOI:** 10.3389/fcvm.2025.1730122

**Published:** 2026-01-16

**Authors:** Mengqi Zhao, Xiaoya Zhang, Enrui Zhang, Junxiang Pan, Yongqiang Jin, Lianyi Wang

**Affiliations:** 1 Heart Center, The First Hospital of Tsinghua University, School of Clinical Medicine, Tsinghua University, Beijing, China; 2 Heart Center, The First Hospital of Tsinghua University, Beijing, China

**Keywords:** diffuse pulmonary arteriovenous malformations, Glenn, total cavopulmonary connection, single-ventricle physiology, congenital heart disease

## Abstract

**Background:**

Diffuse pulmonary arteriovenous malformations (PAVMs) are a rare but serious complication after the Glenn procedure. Clinically, they typically present with progressive hypoxemia, which can lead to worsening cyanosis, reduced exercise tolerance, and may even progress to heart failure.

**Case summary:**

We report an 8-year-old boy with complex congenital heart disease, including double outlet right ventricle, ventricular septal defect, atrial septal defect, and pulmonary stenosis. He underwent a bidirectional Glenn procedure at 6 months of age and subsequently developed progressive cyanosis. At 5 years old, he presented to our center, where comprehensive evaluation confirmed extensive PAVMs. Consequently, an extracardiac total cavopulmonary connection (TCPC) was performed. During the 3-year postoperative follow-up, the PAVMs completely resolved, oxygen saturation normalized, and clinical symptoms improved markedly.

**Conclusion:**

In patients with a history of Glenn procedure who present with unexplained hypoxemia, the possibility of postoperative PAVMs should be carefully considered. Early diagnosis and timely intervention can significantly improve outcomes. This case highlights the therapeutic value of TCPC in managing this complication.

## 1 Introduction

The Glenn procedure is a standard palliative stage in the Fontan pathway for patients with single-ventricle physiology. Compared with a primary Fontan operation, it significantly reduces perioperative mortality (1). However, with increasing long-term survival, follow-up studies have shown that approximately 25% of patients develop clinically significant pulmonary arteriovenous malformations (PAVMs) after the Glenn procedure, and the risk increases with time (2). PAVMs—defined as abnormal direct communications between the pulmonary arteries and veins—create a right-to-left shunt that leads to hypoxemia. Among these, diffuse PAVMs are particularly rare but severe, resulting in profound cyanosis, hemoptysis, reduced exercise tolerance, and even heart failure (3, 4). The pathogenesis of PAVMs is complex and not yet fully understood. Although transcatheter embolization is effective for isolated lesions, the treatment of diffuse disease remains challenging.

Here, we report a case of diffuse PAVMs in a child with complex congenital heart disease after Glenn palliation. The malformations were successfully reversed by total cavopulmonary connection (TCPC), with marked improvement in oxygenation. This case provides insight into the underlying mechanisms and clinical management of Glenn-associated diffuse PAVMs.

## 2 Case report

### 2.1 Patient information and clinical findings

The patient was a boy diagnosed at 42 days of age with complex congenital heart disease, including double outlet right ventricle (DORV) with noncommitted ventricular septal defect (VSD), D-malposition of the great arteries, pulmonary stenosis, and an atrial septal defect (ASD). At 6 months of age, he underwent a bidirectional Glenn procedure along with ASD enlargement and main pulmonary artery ligation. Postoperatively, his cyanosis improved significantly, and peripheral oxygen saturation (SpO₂) increased from 85% to 90%. However, approximately 6 months later, he developed progressive cyanosis, reduced exercise tolerance, and growth retardation. Local medical evaluation suggested that no further surgical options were available at that time, and the family did not seek further cardiology evaluation thereafter. His hypoxemia continued to worsen over the subsequent years. At 5 years of age, he presented to our center with pronounced central cyanosis and digital clubbing. The SpO₂ ranged from 58% to 69%.

### 2.2 Diagnostic assessment

**Figure 1:**
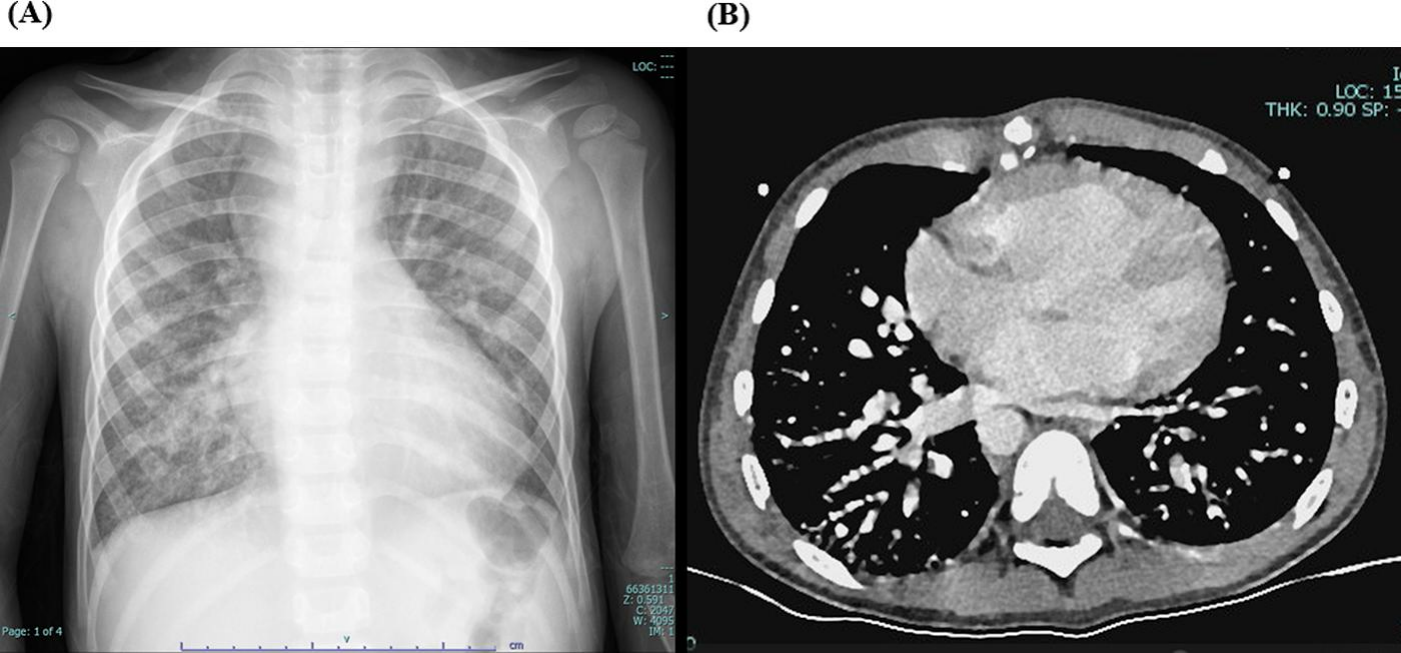
**(A)** preoperative chest X-ray showing increased pulmonary markings. **(B)** Preoperative contrast-enhanced cardiac CT demonstrating simultaneous opacification of pulmonary arteries and veins, with a beaded appearance of the distal pulmonary arteries, more prominent in both lower lobes, consistent with diffuse pulmonary arteriovenous malformations (PAVMs).

**Figure 2:**
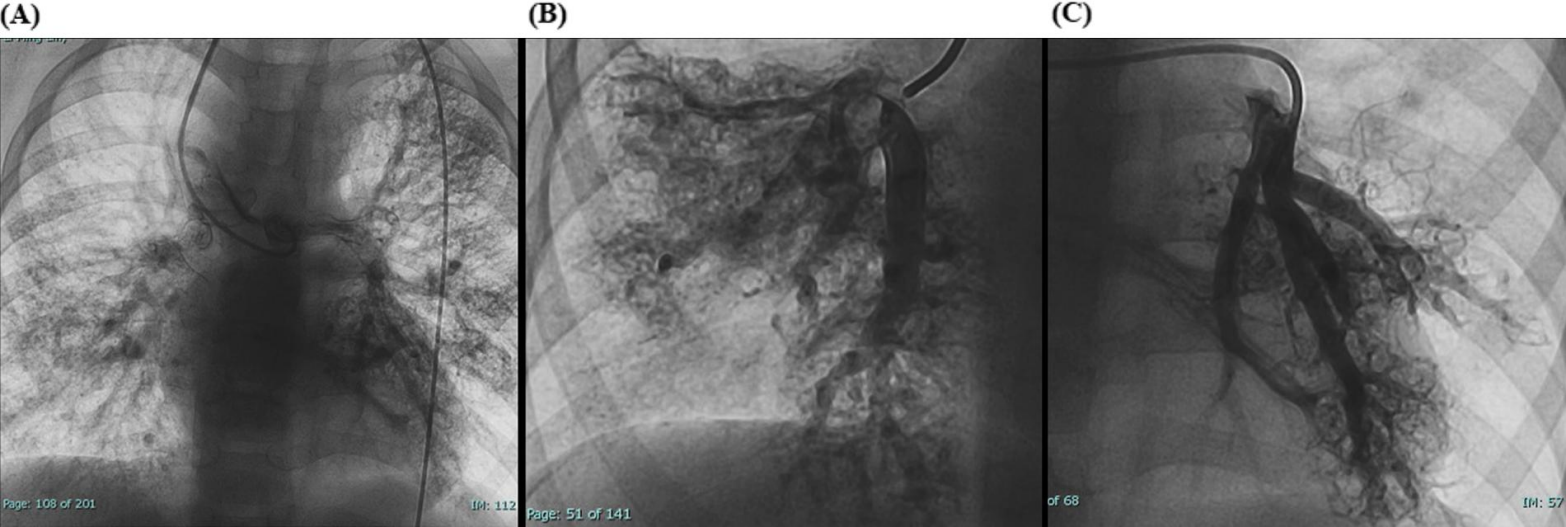
**(A)** Preoperative cardiac catheterization showing diffuse bilateral pulmonary arteriovenous malformations (PAVMs). **(B,C)** Local cardiac catheterization angiographic views showing PAVMs more pronounced in the lower lobes.

Laboratory tests showed hemoglobin of 225 g/L and hematocrit of 73.3%. Arterial blood gas analysis revealed a partial pressure of oxygen (PO₂) of 30.7 mmHg and oxygen saturation (SO₂) of 58.1%. Chest X-ray showed cardiomegaly (cardiothoracic ratio 0.6) and increased pulmonary markings (Figure 1A). Echocardiography revealed absence of antegrade pulmonary artery flow; a 23 mm VSD located beneath the pulmonary valve with bidirectional shunting; a 23 mm ASD with bidirectional shunt; and a patent Glenn anastomosis. Contrast-enhanced CT showed ASD and VSD, absence of the pulmonary valve and main pulmonary artery, confluence of the right and left pulmonary arteries, reduced pulmonary blood flow, and extensive PAVMs in both lungs—most prominent in the lower lobes (Figure 1B). The measured McGoon ratio was 1.857. Cardiac catheterization confirmed absence of antegrade pulmonary artery flow, with well-developed right and left pulmonary arteries. The distal pulmonary arteries and pulmonary venous branches were markedly dilated, with some showing a granular or “string-of-beads” appearance (Figure 2). Pulmonary circulation time was significantly shortened, indicating substantial right-to-left intrapulmonary shunting, consistent with diffuse PAVMs. The lower lobes were most severely involved. Pulmonary artery pressure was 17/9/13 mmHg.

### 2.3 Surgical treatment

A median sternotomy was performed, and cardiopulmonary bypass was instituted. Intraoperative assessment showed that the VSD was located in the inflow tract and was anatomically unsuitable for intracardiac tunnel construction, rendering biventricular repair unfeasible. Therefore, an extracardiac TCPC was undertaken. An 18-mm Gore-Tex conduit was connected from the inferior vena cava to the right pulmonary artery to establish a complete cavopulmonary pathway. Immediate postoperative SpO₂ was about 75%.

The patient remained in the intensive care unit for 8 days and was discharged after a total postoperative hospital stay of 19 days. Following intensive supportive care, his condition gradually stabilized, and he recovered uneventfully.

### 2.4 Follow-up and outcomes

**Figure 3:**
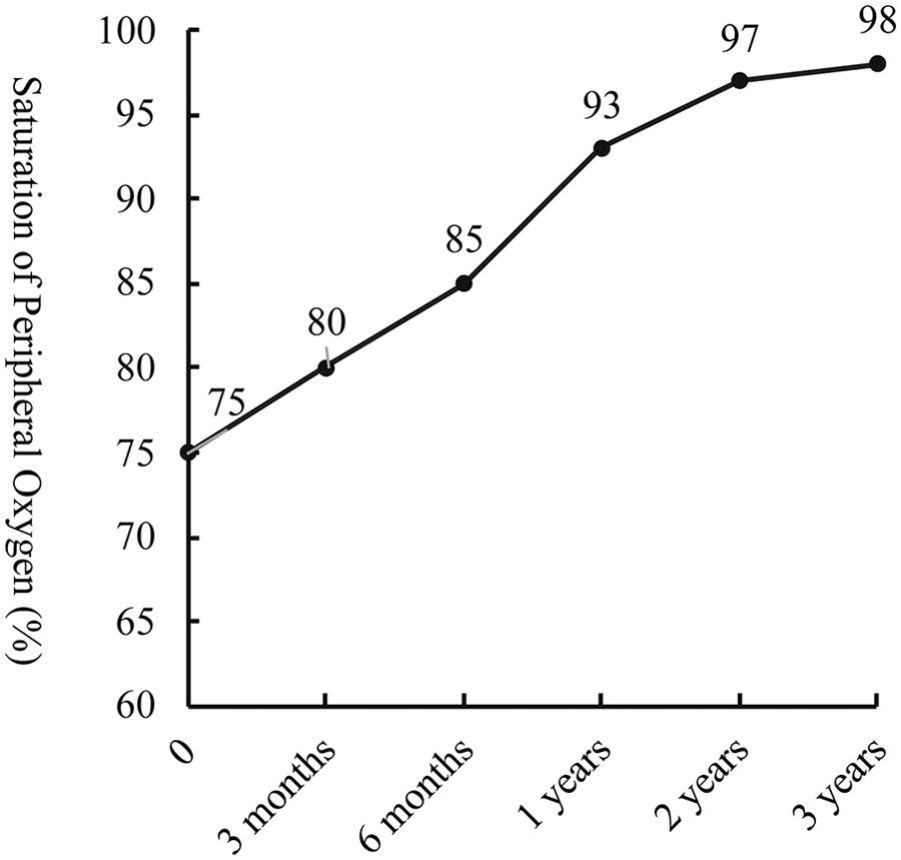
Follow-up trend of saturation of peripheral oxygen (SpO₂) after total cavopulmonary connection surgery (TCPC).

**Figure 4:**
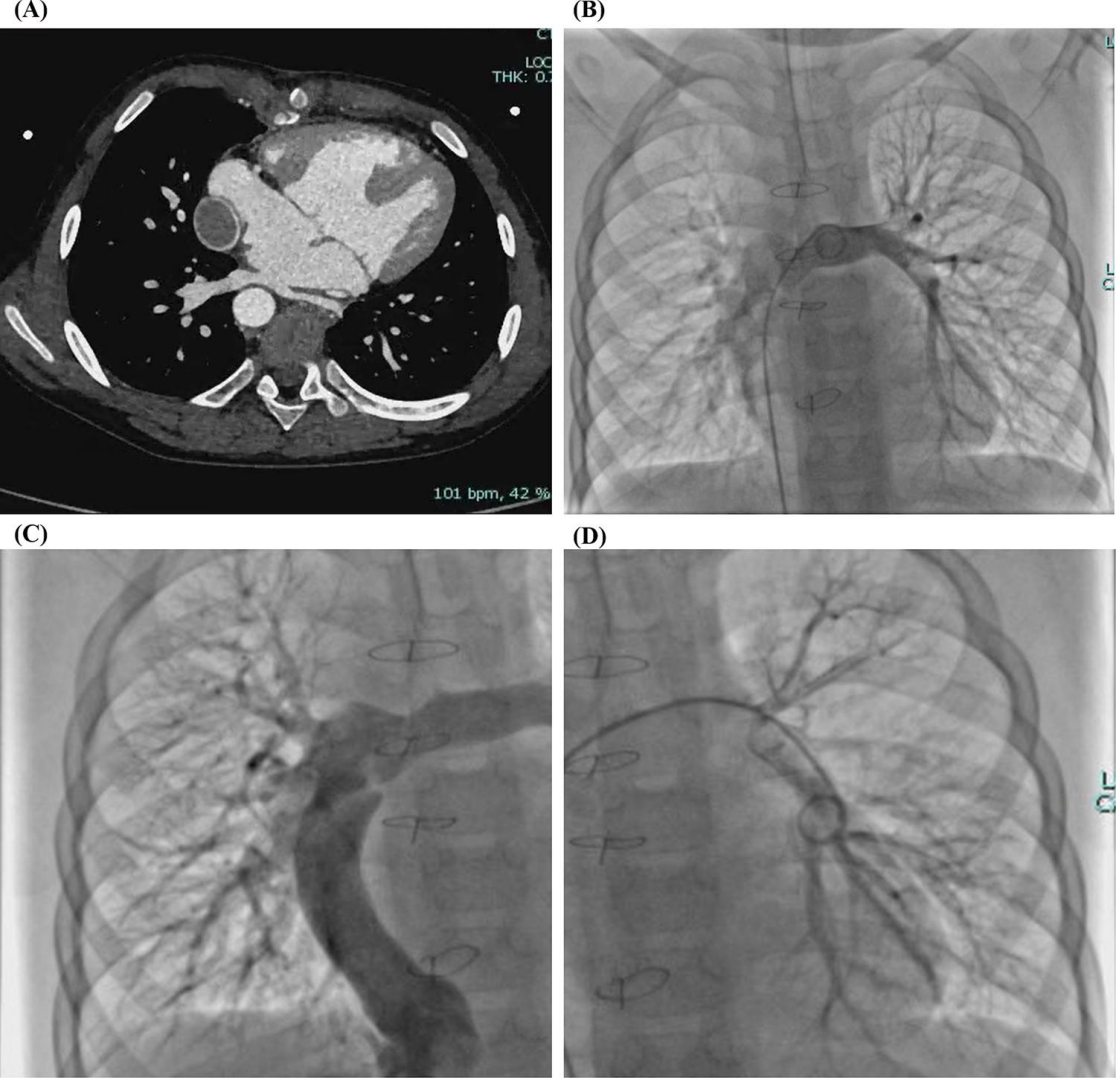
**(A)** Contrast-enhanced cardiac CT after total cavopulmonary connection (TCPC) confirming complete disappearance of the bilateral diffuse pulmonary arteriovenous malformations (PAVMs). **(B–D)** Cardiac catheterization after TCPC demonstrating complete resolution of bilateral diffuse PAVMs.

During a 3-year follow-up period, the SpO₂ gradually increased and stabilized at 97%–100% (Figure 3). Exercise tolerance improved markedly, the lips became pink, and digital clubbing resolved. Hemoglobin decreased to 127 g/L. Arterial blood gas analysis revealed a PaO₂ of 78.6 mmHg and an SO₂ of 95.8%. Contrast-enhanced cardiac CT demonstrated complete disappearance of the previously diffuse bilateral PAVMs (Figure 4A). Repeat cardiac catheterization confirmed unobstructed conduit flow and well-developed pulmonary vasculature. The previously extensive bilateral PAVMs had completely resolved, pulmonary circulation time returned to normal, and pulmonary artery pressures were 13/11/12 mmHg (Figure 4B–D).

## 3 Discussion

Diffuse PAVMs are a rare but serious complication in patients with single-ventricle physiology following the Glenn procedure. The fundamental pathology involves abnormal communications between the pulmonary arteries and veins, resulting in right-to-left shunting and reduced arterial oxygen content. This mechanism represents one of the major causes of progressive cyanosis and hypoxemia in the late postoperative period (4). In the present case, the patient developed progressive cyanosis and decreased exercise tolerance after the bidirectional Glenn procedure, and was diagnosed with diffuse PAVMs at the age of five, consistent with the typical clinical course of this complication.

Although the exact mechanism of PAVM formation remains incompletely understood, the most widely accepted explanation is the “hepatic factor hypothesis”. In the Glenn circulation, pulmonary blood flow derives entirely from the superior vena cava, excluding the inferior vena cava and hepatic venous return. The absence of hepatic venous effluent may result in loss of protective factors and subsequent pulmonary vascular dysregulation (5). Clinical evidence suggests that after a classic unilateral Glenn shunt, PAVMs commonly develop in the ipsilateral lung, especially in the overperfused lower lobes (6). Bilateral diffuse PAVMs are typically seen when both lungs lack hepatic venous perfusion (7). Similarly, PAVMs frequently occur after the Kawashima procedure when hepatic veins drain directly into the atrium rather than the pulmonary arteries (3). Restoration of hepatic venous flow to the pulmonary circulation—most commonly through completion of the TCPC—can lead to gradual regression of PAVMs (2). The significant improvement observed in this patient following TCPC supports this mechanism.

The nature and action of the so-called hepatic factor remain to be fully elucidated. It has been proposed that PAVMs may result from an imbalance between proangiogenic and antiangiogenic mediators within the pulmonary circulation, leading to abnormal vascular proliferation and direct arteriovenous communications (8). Non-pulsatile flow, uneven perfusion distribution, and specific biochemical signaling from the superior vena cava may also contribute (9). On a molecular level, miR-25-3p has been shown to promote angiogenesis via the Akt/mTOR and HIF-1α/VEGF pathways (10), while dysregulation of the TGF-β signaling pathway may be involved in PAVM formation, particularly in complex cardiac malformations such as heterotaxy syndrome (2).

For diagnosis, contrast-enhanced echocardiography is the preferred screening tool, where microbubbles appearing in the left heart 3–6 cardiac cycles after injection indicate intrapulmonary right-to-left shunting. Contrast-enhanced CT angiography (CTA) provides detailed visualization of the location, distribution, and size of PAVMs, while pulmonary angiography remains the gold standard for definitive diagnosis and potential interventional therapy. Integrating data from pulse oximetry, imaging studies, perfusion scans, and contrast echocardiography can improve diagnostic accuracy.

The cornerstone of PAVM management is restoration of hepatic venous blood flow to the pulmonary circulation. For isolated or focal lesions, transcatheter embolization is an effective and minimally invasive treatment. However, diffuse or multifocal PAVMs remain challenging to manage (7). Surgical restoration of hepatic venous flow, most commonly through TCPC completion, remains the most effective strategy to reverse extensive PAVMs and improve oxygenation. Brown et al. reported that a delay exceeding two years between the Kawashima procedure and hepatic venous inclusion was an independent risk factor for PAVM development (3), emphasizing the importance of timely reintroduction of hepatic venous return.

In selected patients, partial preservation of antegrade pulmonary blood flow (APBF) during the Glenn procedure may help prevent PAVM formation. Behrend et al. (11) found that maintaining APBF in high-risk patients could sustain oxygenation, promote pulmonary artery growth, and improve outcomes after TCPC completion. However, excessive APBF may increase ventricular volume load and the risk of pleural effusions. Thus, individualized evaluation based on anatomic and physiologic characteristics is required to balance its potential benefits and risks.

## 4 Conclusions

In summary, although diffuse PAVMs are a rare but serious complication following the Glenn procedure, their reversibility provides an opportunity for effective intervention. This case highlights the importance of vigilance for PAVMs in patients presenting with progressive hypoxemia after Glenn palliation. Early restoration of hepatic venous flow to the pulmonary circulation—or, when appropriate, preservation of APBF—may prevent or reverse PAVM development. Prompt recognition and timely management are crucial to improving long-term outcomes.

## Data Availability

The original contributions presented in the study are included in the article, further inquiries can be directed to the corresponding author.
